# Early Metabolomic and Immunologic Biomarkers as Prognostic Indicators for COVID-19

**DOI:** 10.3390/metabo14070380

**Published:** 2024-07-09

**Authors:** Zigui Chen, Erik Fung, Chun-Kwok Wong, Lowell Ling, Grace Lui, Christopher K. C. Lai, Rita W. Y. Ng, Ryan K. H. Sze, Wendy C. S. Ho, David S. C. Hui, Paul K. S. Chan

**Affiliations:** 1 Department of Microbiology, Faculty of Medicine, The Chinese University of Hong Kong, Hong Kong SAR 999077, China; zigui.chen@cuhk.edu.hk (Z.C.); chris.kclai@cuhk.edu.hk (C.K.C.L.); ritang@cuhk.edu.hk (R.W.Y.N.); skh405@ha.org.hk (R.K.H.S.); cc_wendy@cuhk.edu.hk (W.C.S.H.); 2 Cardiovascular Science Center and Division of Cardiology, School of Medicine, The Chinese University of Hong Kong, Shenzhen 518172, China; e.fung@cuhk.edu.hk; 3 School of Biomedical Sciences, Faculty of Medicine, The Chinese University of Hong Kong, Hong Kong SAR 999077, China; 4 Department of Epidemiology and Biostatistics, School of Public Health, Imperial College London, White City, London SW7 2AZ, UK; 5 Department of Chemical Pathology, Faculty of Medicine, The Chinese University of Hong Kong, Hong Kong SAR 999077, China; ck-wong@cuhk.edu.hk; 6 Department of Anaesthesia and Intensive Care, Faculty of Medicine, The Chinese University of Hong Kong, Hong Kong SAR 999077, China; lowell.ling@cuhk.edu.hk; 7 Department of Medicine and Therapeutics, Faculty of Medicine, The Chinese University of Hong Kong, Hong Kong SAR 999077, China; gracelui@cuhk.edu.hk (G.L.); dschui@cuhk.edu.hk (D.S.C.H.)

**Keywords:** metabolome, chemokine, cytokine, COVID, coronavirus, SARS-CoV-2, severity, biomarker

## Abstract

This prospective study in Hong Kong aimed at identifying prognostic metabolomic and immunologic biomarkers for Coronavirus Disease 2019 (COVID-19). We examined 327 patients, mean age 55 (19–89) years, in whom 33.6% were infected with Omicron and 66.4% were infected with earlier variants. The effect size of disease severity on metabolome outweighed others including age, gender, peak C-reactive protein (CRP), vitamin D and peak viral levels. Sixty-five metabolites demonstrated strong associations and the majority (54, 83.1%) were downregulated in severe disease (z score: −3.30 to −8.61). Ten cytokines/chemokines demonstrated strong associations (*p* < 0.001), and all were upregulated in severe disease. Multiple pairs of metabolomic/immunologic biomarkers showed significant correlations. Fourteen metabolites had the area under the receiver operating characteristic curve (AUC) > 0.8, suggesting a high predictive value. Three metabolites carried high sensitivity for severe disease: triglycerides in medium high-density lipoprotein (MHDL) (sensitivity: 0.94), free cholesterol-to-total lipids ratio in very small very-low-density lipoprotein (VLDL) (0.93), cholesteryl esters-to-total lipids ratio in chylomicrons and extremely large VLDL (0.92);whereas metabolites with the highest specificity were creatinine (specificity: 0.94), phospholipids in large VLDL (0.94) and triglycerides-to-total lipids ratio in large VLDL (0.93). Five cytokines/chemokines, namely, interleukin (IL)-6, IL-18, IL-10, macrophage inflammatory protein (MIP)-1b and tumour necrosis factor (TNF)-a, had AUC > 0.8. In conclusion, we demonstrated a tight interaction and prognostic potential of metabolomic and immunologic biomarkers enabling an outcome-based patient stratification.

## 1. Introduction

Since the emergence of the Severe Acute Respiratory Syndrome Coronavirus 2 (SARS-CoV-2) in late 2019, more than 775 million infected cases and 7 million deaths have been reported to the WHO as of April 2024. Currently, the virus is still evolving and actively spreading around the globe [1].

SARS-CoV-2 infection starts primarily in the respiratory tract and can progress to a life-threatening systemic illness with organ failure. However, the majority of cases are asymptomatic or present with mild upper respiratory tract symptoms, with less than 5% becoming critical or fatal [2,3]. While age and co-morbidity are strongly associated with severe disease, the outcome of infection can be difficult to predict [4]. Early biomarkers with a high predictive value for clinical outcome would be valuable in the triage of patients for the early appropriate and cost-effective management of the infection. Previous genomic, transcriptomic and proteomic studies have depicted and delineated disease mechanisms, and opened a horizon to explore potential biomarkers to predict the outcome of Coronavirus Disease 2019 (COVID-19) [5,6,7,8,9].

Viruses use numerous small molecules of the infected cell as sources of energy for replication, and building blocks for the progeny viruses. Therefore, studying the cell metabolome or the subsequent metabolomic changes in body fluid can reflect the state of viral activity, host cell pathology and response, providing insights into the outcome of viral infection. Nuclear magnetic resonance (NMR)-based metabolomics is an attractive approach to characterize the complex host–virus interaction. This approach has shed light on the disease mechanism of COVID-19, and revealed the determinants of biochemical pathways from the initial steps of infection to its progression to recovery or fatal disease [10].

In this study, we applied proton (^1^H)-NMR-based targeted metabolomics to delineate the blood metabolome of COVID-19 patients who eventually progressed to varying degrees of severity. We also characterized the concurrent changes in circulatory immunologic biomarkers to explore the potential of using early metabolomic and immunologic biomarkers as a prognostic indicator for the outcome of COVID-19.

## 2. Materials and Methods

### 2.1. Subject Recruitment

This prospective study was conducted from February 2020 to April 2022 covering the first five major waves of COVID-19 in Hong Kong. Adult patients admitted to the Prince of Wales Hospital, the teaching hospital of the Chinese University of Hong Kong, with PCR-confirmed SARS-CoV-2 infection were invited. All study subjects provided a written informed consent and the study was approved by the Joint Chinese University of Hong Kong–New Territories East Cluster Clinical Research Ethics Committee.

### 2.2. Sampling and Investigations

The first EDTA blood sample collected from all participants soon after admission to hospital was used for metabolomic and immunologic investigations pertaining to this study. Samples were cooled immediately with ice and delivered to the laboratory. Plasma was separated by centrifugation (2000× *g* for 10 min) at 4 °C and stored at −70 °C until analysis.

Plasma samples were heat-inactivated at 56 °C for 30 min before shipping for targeted metabolomic analysis using ^1^H-NMR spectroscopy (Nightingale Health, Helsinki, Finland). A total of 170 metabolites were quantified simultaneously, including absolute concentrations of lipoproteins and lipid contents within 14 lipoprotein subclasses, conventional lipids and low-molecular-weight metabolites (LMWMs), including amino acids, ketone bodies, glycolysis-related metabolites and glycoprotein acetyls (GlycA) as well as ratios of fatty acids to total fatty acids. The ^1^H-NMR pipeline and platform have been used and extensively applied in large-scale epidemiological studies, and well described previously [11,12,13,14].

Plasma 25-OH vitamin D level was measured using an enzyme immunoassay (25-OH vitamin D, Euroimmun, UK), and a cut-off level of 20 ng/mL was used to define the “high-“ and “low-“ level groups for the purpose of comparison in this study [15].

Serial upper respiratory tract specimens including nasopharyngeal swabs and deep throat saliva were collected during the first week of hospitalization for real-time PCR as described previously [16]. The peak viral load was defined as the one with the lowest threshold cycle (Ct) among specimens available for testing.

Plasma concentrations of 40 cytokines/chemokines were measured using the Milliplex human cytokine multiplex assay using the Bio-plex 200 System (Bio-Rad Laboratories, Inc., Hercules, CA, USA). The panel of cytokines/chemokines included sCD40L, epidermal growth factor (EGF), eotaxin/CCL11, fibroblast growth factor-2 (FGF-2), Fms-like tyrosine kinase 3 ligand (FLT-3L), fractalkine, granulocyte colony-stimulating factor (G-CSF), granulocyte–macrophage colony-stimulating factor (GM-CSF), growth-regulated oncogene (GRO)-a, interferon (IFN)-a2, IFN-g, Interleukin (IL)-1a, IL-1b, IL-1RA, IL-2, IL-3, IL-4, IL-5,IL-6, IL-7, IL-8, IL-9, IL-10, IL-12 (p40), IL-12 (p70), IL-13, IL-15, IL-17A, IL-18, IP-10, monocyte chemoattractant protein (MCP)-1, MCP-3, macrophage-derived chemokine (MDC) (CCL22), monokine induced by gamma interferon (MIG)/CXCL9, macrophage inflammatory protein (MIP)-1a, MIP-1b, transforming growth factor (TGF-a), tumour necrosis factor (TNF)-a, TNF-b and vascular endothelial growth factor (VEGF).

### 2.3. Analysis

For the purpose of analysis in this study, the clinical outcome of COVID-19 was defined according to the WHO criteria [17]. Briefly, critical cases included those who developed acute respiratory distress syndrome, sepsis, septic shock or required life-sustaining treatments such as vasopressor, mechanical ventilation or extracorporeal membrane oxygenation (ECMO). Severe cases included those with saturation of peripheral oxygen < 90% on room air, respiratory rate > 30 or signs of severe respiratory distress. Those without signs of severe or critical disease were classified as “non-severe”. When appropriate, “non-severe” cases were further categorized into “moderate”: those with clinical signs of pneumonia, but no signs of severe pneumonia; and “mild”: those without signs of pneumonia. Fatal disease was defined as any death that was primarily due to COVID-19.

The odds ratios (ORs) between disease severity and demographic variables and laboratory findings, including sex, age, peak C-reactive protein (CRP) level, plasma 25-OH vitamin D level and peak respiratory viral load, were examined using both univariate and multivariate regression models.

Clustering between metabolomic biomarkers and disease severity was evaluated using principal coordinate analysis (PCoA) based on Bray–Curtis distance metrics. The beta diversity between different severity groups was analyzed using permutational multivariate analysis of variance (PERMANOVA) with 9999 permutations using the “vegan” package in R (version 4.3.1); (R Core Team (2023); R: A Language and Environment for Statistical Computing; R Foundation for Statistical Computing, Vienna, Austria).

The Jonckheere–Terpstra test was applied to assess ordered associations of metabolomic and immunologic biomarkers with clinical features. This test was conducted using the “JonckheereTest” function from the PMCMRplus package in R. Correlations between changes in cytokine/chemokine and metabolite levels were determined using Spearman’s correlation, calculated using the “cor.test” function from the stats package in R. The correlations were visualized in heat maps, displaying the rho values.

The sensitivity and specificity of metabolomic and immunologic biomarkers for predicting severe COVID-19 were examined using the receiver operating characteristic (ROC) analysis from the pROC package in R. The area under the ROC curve (AUC) and the optimal cutoff values determining specificity and sensitivity were identified using the default parameters. Graphical illustrations, including the generation of heat maps, correlations, boxplots and ROC curves, were created using the ggplot2 package in R.

## 3. Results

A total of 327 subjects aged 19–89 (mean [standard deviation]: 55 [17]) years with 33.6% being male were recruited (Table S1). The key demographic and clinical characteristics are shown in Table 1. Altogether, 30.3% (99/327) had severe disease (9 fatal, 55 critical, and 35 severe pneumonia), and 69.7% (228/327) had non-severe disease (14 asymptomatic, 123 mild and 91 moderate). Of the 327 patients, 217 (66.4%) were infected with the authentic virus or earlier variants circulated before the emergence of the Omicron lineage; whereas 110 (33.6%) were infected with the Omicron variant, mainly BA.2, during the fifth wave of early 2022 in Hong Kong.

**Figure 1:**
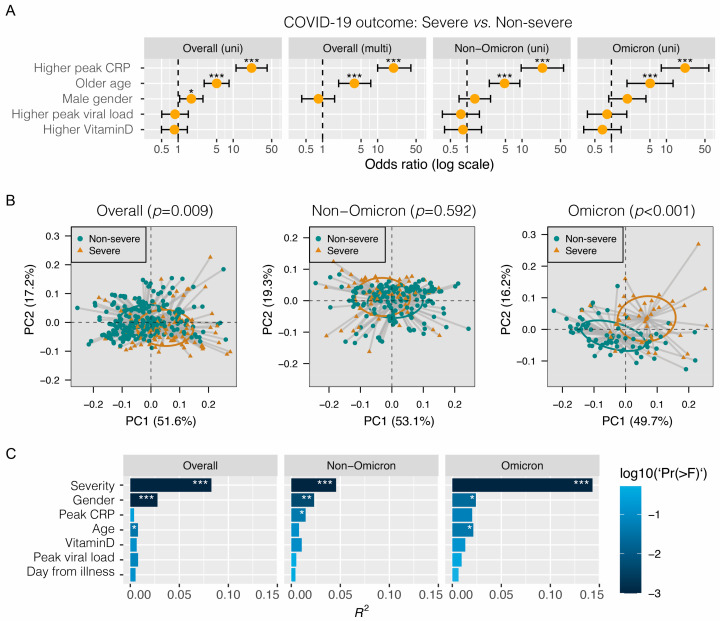
Association between severity of COVID-19 and demographic, metabolomic and other biomarkers. (**A**) Univariate (uni) and multivariate (multi) regression analyses. Severe disease includes fatal, critical and severe pneumonia according to WHO criteria. Non-severe disease includes moderate, mild and asymptomatic. (**B**) Principal coordinate analysis based on Bray–Curtis distance metrics inferred from metabolomic profiles. Beta diversity between severe and non-severe groups was evaluated using permutational multivariate analysis of variance (PERMANOVA) with 9999 permutations. (**C**) Effect size (R2 value) of variables on changes of the metabolomic profile in the surveyed samples. Disease severity was adjusted for covariates including gender (female vs. male), age (≤60 vs. >60 years), peak CRP (≤30 vs. >30 mg/L), peak viral load (Ct ≤ 20 vs. >20) and 25-OH vitamin D (≤20 vs. >20 ng/mL) in adonis2. * *p* < 0.05; ** *p* < 0.01; *** *p* < 0.001.

Firstly, we examined the associations between disease severity and key demographic variables and laboratory findings. As shown in Figure 1A, older age (>60 years) and higher peak CRP (>30 mg/L) were significantly associated with severe disease in the overall, non-Omicron and Omicron groups (*p* < 0.001 in multivariate comparisons for all three patient groups). Male gender exhibited a significant association with severe disease in univariate analysis of the overall group (*p* < 0.05), but was not significant upon multivariate analysis. Of note, upper respiratory tract peak viral load and plasma 25-OH vitamin D level did not carry any significant associations with disease severity (Figure 1A).

As shown in Figure 1B, a more segregated metabolomic profile distinguishing severe (severe pneumonia, critical and fatal) from non-severe patients (asymptomatic, mild and moderate pneumonia) was observed for the Omicron group (*p* < 0.001), compared with the non-Omicron group (*p* = 0.592). The effect size of disease severity on metabolomic profile outweighed other factors examined including age, gender, peak CRP, vitamin D level, peak viral load and day from illness onset, both for the non-Omicron (R^2^: 0.046, *p* < 0.001) and Omicron (R2: 0.143, *p* < 0.001) groups (Figure 1C). In particular, the effect size of disease severity surpassed other factors as being greatest for the Omicron group. Of note, gender also had a strong effect on the metabolomic profile particularly in the non-Omicron group.

**Figure 2:**
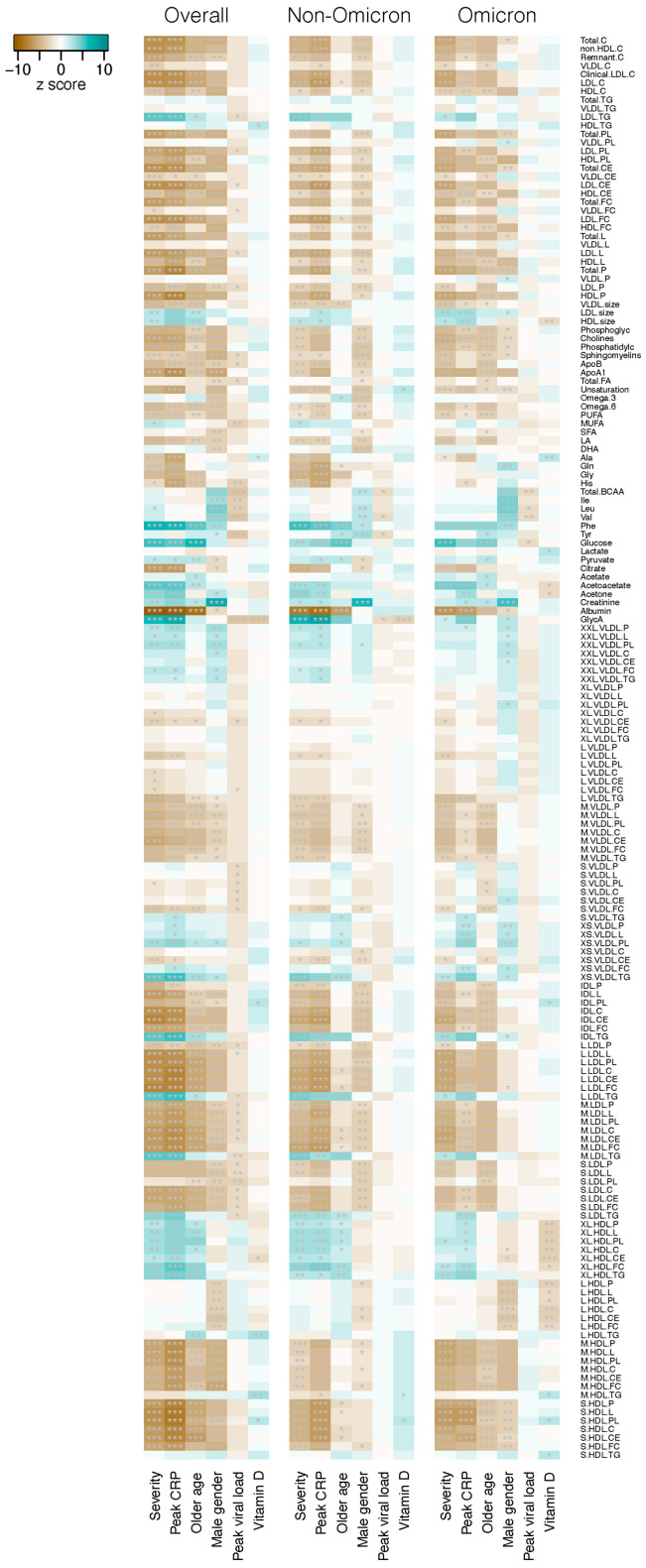
Association of plasma metabolite levels with COVID-19 severity and other variables. The z-scores from the Jonckheere–Terpstra test for ordered alternatives using the JonckheereTest in the PMCMRplus R package are shown in the heat map. * *p* < 0.05; ** *p* < 0.01; *** *p* < 0.001. COVID-19 severity was classified into six categories: from asymptomatic, mild, moderate, severe, critical to fatal. Peak CRP: from low- to high-level values. Age: from young to old age years. Gender: from female to male. Peak viral load: from high- to low-Ct values. 25-OH vitamin D: from low- to high-level values. The full names of the metabolites are given in Table S2. Left panel: all patients (*n* = 327), middle panel: non-Omicron patients (*n* = 217) recruited between 2020 and 2021 before the emergence of Omicron variants, right panel: Omicron patients (*n* = 110) recruited after January 2022, and were all infected with Omicron variants, mainly BA.2.

Secondly, we examined the associations between plasma concentration of individual metabolites and disease severity in association with patient age, gender, peak CRP, peak viral load and 25-OH vitamin D level. As shown in Figure 2 and Table S2, a large number of metabolites, including cholesterol, triglycerides, phospholipids, cholesteryl esters, lipids, lipoproteins, apolipoproteins, fatty acids, amino acids, ketone bodies and metabolites related to glycolysis, fluid balance and inflammation, were found to be strongly associated (Jonckheere–Terpstra ordered association test, *p* < 0.001) with disease severity. Of the 65 metabolites showing a highly significant association among the non-Omicron group, the majority (54, 83.1%) was downregulated in severe disease with z scores ranging from −3.30 to −8.61. Similar findings were observed among the Omicron group, in which 73 (96.1%) of 76 metabolites with highly significant association were downregulated in severe disease, with z scores ranging from −3.34 to −7.11.

The majority of the metabolites also demonstrated a strong significant negative association with CRP levels, whereas such pattern was not observed for peak viral load and 25-OH vitamin D levels (Figure 2).

**Figure 3:**
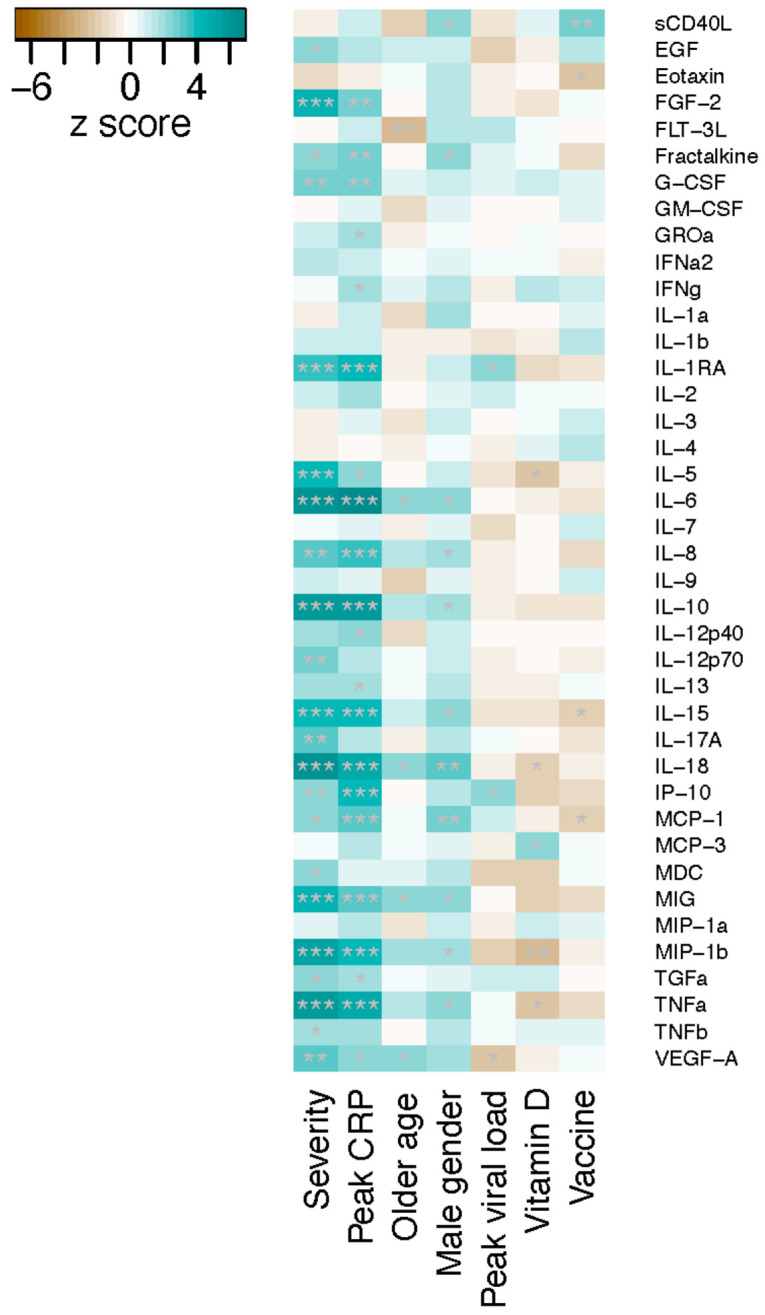
Association of plasma cytokine/chemokine levels with COVID-19 severity and other variables in patients infected with the Omicron variant. The z-scores from the Jonckheere–Terpstra test for ordered alternatives using the JonckheereTest in the PMCMRplus R package are shown in the heat map. * *p* < 0.05; ** *p* < 0.01; *** *p* < 0.001. COVID-19 severity was classified into six categories ranging from asymptomatic, mild, moderate, severe, critical to death. Peak CRP: from low- to high-level values. Age: from young to old age years. Gender: from female to male. Peak viral load: from high- to low-Ct values. 25-OH vitamin D: from low- to high-level values. Vaccine: from 0, 1, 2 to 3 doses.

We then examined the immunologic biomarkers. Plasma concentrations of 40 cytokines/chemokines were measured for the Omicron group (*n* = 110), and 10 cytokines/chemokines were found to have a highly significant association with disease severity (Jonckheere–Terpstra test, *p* < 0.001) (Figure 3, Table S3). All these cytokines/chemokines, including fibroblast growth factor (FGF)-2, IL-1RA, IL-5, IL-6, IL-10, IL-15, IL-18, MIG, MIP-1b and TNFa, were upregulated in severe disease. Similar to the observation on metabolites, cytokine/chemokine profiles also displayed a close association with peak CRP levels, but not with other variables examined including age, gender, viral load, 25-OH vitamin D and vaccine exposure (Figure 3).

**Figure 4:**
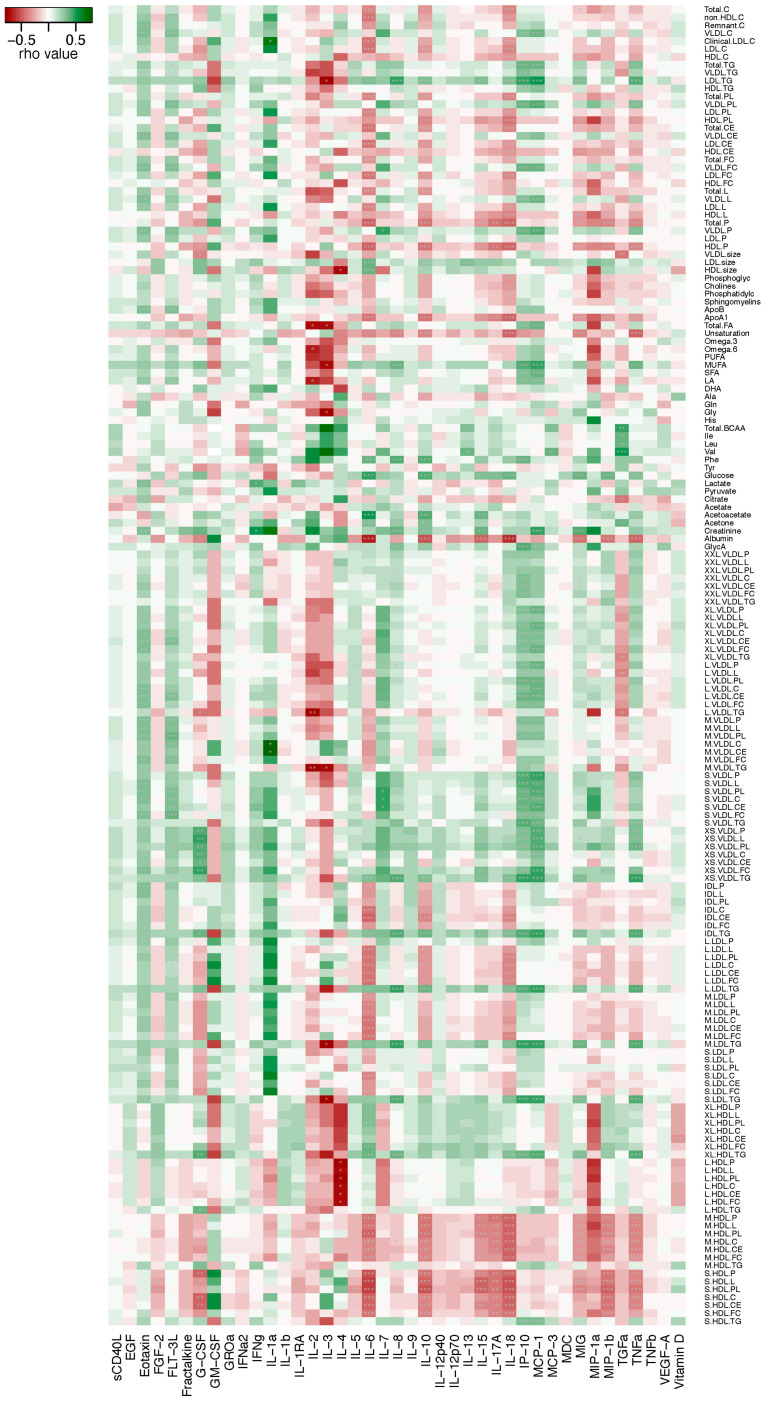
Correlation between plasma metabolite and cytokine/chemokine levels. Only the Omicron group with both cytokine/chemokine and metabolomic data available are included. The rho values from the Spearman’s correlation test using the cor.test in the Stats R are shown in the heat map. * *p* < 0.05; ** *p* < 0.01; *** *p* < 0.001. 25-OH vitamin D was included as a reference.

Figure 4 shows the correlation between metabolite and cytokine/chemokine levels among the Omicron group, revealing multiple metabolite and cytokine/chemokine pairs with strong positive or negative associations were revealed (Figure 4, Table S4). For instance, albumin level had a significant negative correlation with multiple cytokines/chemokines including IL-6, IL-10, IL-15, IL-18, MIG, MIP-1b and TNFa, whereas creatinine level showed a significant positive correlation with IL-1a, IL-8, MCP-1 and MIG. While IL-1a level had a significant positive correlation with cholesterol and cholesteryl esters in medium VLDL (M.VLDL.C and M.VLDL.CE), IL-4 level had a significant negative correlation with total lipids, phospholipids, cholesterol, cholesteryl esters and free cholesterol in very large HDL (XL.HDL.L/PL/C/CE/FC) as well as with the concentration of very large HDL particles (XL.HDL.P). Details of the Spearman’s analysis with the corresponding rho and *p* values are shown in Table S4.

**Figure 5:**
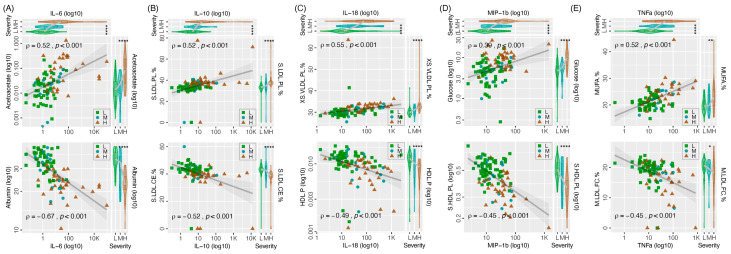
Scatter plots showing representative correlations between plasma metabolite and cytokine/chemokine levels. Only the Omicron group with both cytokine/chemokine and metabolomic data available are included. (**A**) Positive and negative correlations of IL-6 with acetoacetate and albumin, respectively. (**B**) Positive and negative correlations of IL-10 with phospholipids-to-total lipids ratio in small low-density lipoproteins (S.LDL.PL.%) and cholesteryl esters-to-total lipids ratio in small low-density lipoproteins (S.LDL.CE.%), respectively. (**C**) Positive and negative correlations of IL-18 with phospholipids-to-total lipids ratio in very small very low-density lipoproteins (XS.VLDL.PL.%) and concentration of high-density lipoprotein particles (HDL.P), respectively. (**D**) Positive and negative correlations of MIP-1b with glucose and phospholipids in small high-density lipoproteins (S.HDL.PL), respectively. (**E**) Positive and negative correlations of TNFa with ratio of monounsaturated fatty acids to total fatty acids (MUFA.%) and free cholesterol-to-total lipids ratio in medium lipoproteins (M.LDL.FC.%), respectively. Metabolite and cytokine/chemokine levels according to disease severity (L: asymptomatic and mild; M: moderate and severe; H: critical and fatal) are shown in the top and right panels of each figure. The rho values from the Spearman’s correlation test were calculated using the cor.test in the Stats R. * *p* < 0.05; ** *p* < 0.01; **** *p* < 0.0001.

The top five cytokines/chemokines showing a strong association with disease severity were selected to further exhibit the correlation between metabolic and immunologic biomarkers (Figure 5). For each cytokine/chemokine, a metabolite with positive correlation and another metabolite with negative correlation with the interested cytokine/chemokine were selected as examples to illustrate their correlation.

**Figure 6:**
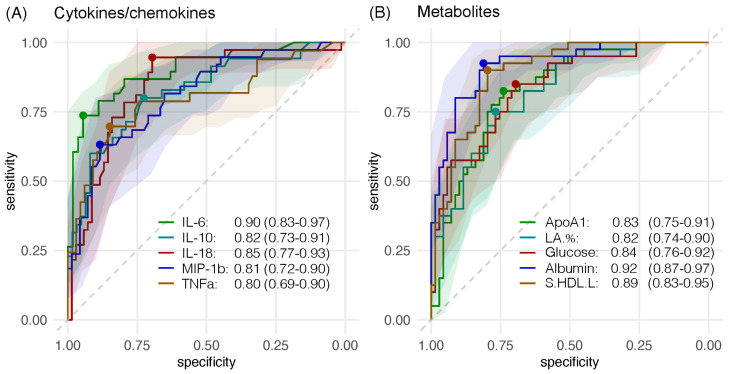
ROC plots of sensitivity and specificity on disease severity for representative cytokines/chemokines and metabolites. Only the Omicron group with both cytokine/chemokine and metabolomic data available are included. (**A**) Five cytokines/chemokines with highest AUC are shown. (**B**) Representative metabolites from five subclasses are shown. The dots represent the best threshold between the specificity and the sensitivity. ApoA1: apolipoprotein A1. LA.%: ratio of linoleic acid to total fatty acids. S.HDL.L: total lipids in small HDL.

The accuracy of metabolomic and immunologic biomarkers to predict disease severity was evaluated by the ROC analysis. The ROC plots of representative metabolomic and immunologic biomarkers are shown in Figure 6. Of the 170 metabolites examined, 14 showed an AUC of greater than 0.8 suggesting a high predictive value (Table S2). These highly predictive metabolomic biomarkers included albumin (specificity: 0.84, sensitivity: 0.76), phospholipids-to-total lipids ratio in very small very low-density lipoprotein (XS.VLDL.PL.%) (0.88, 0.73), cholesterol-to-total lipids ratio in intermediate-density lipoprotein (IDL.C.%) (0.76, 0.79), cholesteryl esters-to-total lipids ratio in IDL (IDL.CE.%) (0.81, 0.75), triglycerides-to-total lipids ratio in IDL (IDL.TG.%) (0.75, 0.77), cholesterol-to-total lipids ratio in large LDL (L.LDL.C.%) (0.76, 0.78), cholesteryl esters-to-total lipids ratio in large LDL (L.LDL.CE.%) (0.79, 0.75), triglycerides-to-total lipids ratio in large LDL (L.LDL.TG.%) (0.70, 0.86), cholesterol-to-total lipids ratio in medium LDL (M.LDL.C.%) (0.64, 0.92), cholesteryl esters-to-total lipids ratio in medium LDL (M.LDL.CE.%) (0.78, 0.71), triglycerides-to-total lipids ratio in medium LDL (M.LDL.TG.%) (0.65, 0.91), phospholipids-to-total lipids ratio in small LDL (S.LDL.PL.%) (0.82, 0.71), cholesterol-to-total lipids ratio in small LDL (S.LDL.C.%) (0.76, 0.84) and cholesteryl esters-to-total lipids ratio in small LDL (S.LDL.CE.% (0.86, 0.66).

Furthermore, three metabolites carried high sensitivity for predicting severe disease including triglycerides in medium high-density lipoprotein (M.HDL.TG) (sensitivity: 0.94), free cholesterol-to-total lipids ratio in very small VLDL (XS.VLDL.FC.%) (0.93), cholesteryl esters-to-total lipids ratio in chylomicrons and extremely large VLDL (XXL.VLDL.CE.%) (0.92) (Table S2), whereas the metabolic markers showing highest specificity for predicting severe disease were creatinine (specificity: 0.94), phospholipids in large VLDL (L.VLDL.PL) (0.94) and triglycerides-to-total lipids ratio in large VLDL (0.93) (L.VLDL.TG.%) (Table S2).

For immunologic biomarkers, five of the 40 cytokines/chemokines examined had AUC values greater than 0.8 suggesting a high predictive value for disease severity (Table S3). These included IL-6 (specificity: 0.94, sensitivity: 0.74), IL-18 (0.70, 0.95), IL-10 (0.73, 0.80), MIP-1b (0.88, 0.63) and TNFa (0.85, 0.70). Furthermore, four immunologic biomarkers had a high sensitivity for predicting severe disease, including GM-CSF, IL-3, IL-4 (all with sensitivity: 1.00) and IL-18 (0.95), whereas immunologic markers with high specificity for severe disease included sCD40L (specificity: 1.00), IL-1a (1.00), (MIG (0.99), IP-10 (0.98), IL-6 (0.94), IL-8 (0.94), IL-17A (0.92) and MCP-1 (0.91).

## 4. Discussion

Metabolomics is an important component of systems biology that systematically analyses small metabolites, <1000 Dalton, of various metabolic pathway matrices, intermediates and products. ^1^H-NMR metabolomic analysis can delineate the profile of endogenous small molecule compounds including sugars, organic acids, amino acids, lipids and glycosylation patterns (e.g., GlycA and GlycB) in different health conditions that provide valuable insights into various medical fields including cancer research, drug discovery and nutrition as well as mechanistic study of infectious diseases [18,19].

Previous studies have shown characteristic metabolomic profiles in plasma collected from COVID-19 patients [20]. Signatures of metabolomic alternation have been found to correlate with severe disease in COVID-19 that included downregulation of malate, aspartate, D-xylulose-5-phosphate, guanosine monophosphate (GMP) and carbamoyl phosphate levels [21]. Furthermore, dysregulation in amino acid metabolism including glutamate and tryptophan metabolites [22], alternations in carbohydrate and energy metabolism, tricarboxylic acid (TCA), purine metabolism, polyamines and nicotinamide metabolites have also been reported [23,24].

In line with previous reports, the current study found a strong association between plasma metabolites and the outcome of COVID-19 severity [25,26,27,28,29,30]. In both our patient groups infected respectively with Omicron and older variants, the effect size of disease severity on metabolomic profile was much greater compared with age, gender, peak CRP, vitamin D and peak viral load. This observation further supports the blood metabolome as a valuable source for mining prognostic biomarkers. In fact, we observed that more than one-third of the metabolites examined can be considered as potentially having a strong correlation with disease severity that deserve further evaluation. These potential metabolites were derived from a wide range of classes and subclasses covering different metabolic pathways including cholesterol, triglycerides, phospholipids, cholesteryl esters, lipids, lipoproteins, apolipoproteins, fatty acids, amino acids, ketone bodies and metabolites related to glycolysis, fluid balance and inflammation. We suggest to select a combination of metabolites from different pathways to compose a set of biomarkers for further evaluation on their prognostic value.

For the Omicron cohort, we used the same plasma sample to examine a wide spectrum on cytokines/chemokines. We observed strong positive and negative correlations between dysregulation of certain metabolites and cytokines/chemokines, confirming their tight mechanistic relationship. The metabolic and immunologic reprogramming are potential pathologic mechanisms for COVID-19 progression, where lipid metabolism and lipoprotein(s) could be novel targets of therapeutic intervention [31,32], whereas for prognostic biomarkers, there is a potential to couple these tightly associated metabolomic and immunologic biomarkers to improve disease monitoring and prediction of clinical outcomes.

Based on our ROC analysis, 14 metabolites and 5 cytokines/chemokines could be considered as having a good potential to be prognostic biomarkers to predict severe disease. In addition to AUC values, other advanced technologies, such as artificial intelligence (AI) and machine learning, hold promise for integrating diverse biomarkers to refine predictive models and provide more personalized approaches to patient management in clinical settings. We propose a combinatorial approach where these metabolomic markers provide complementary information that could enhance prognostic accuracy when used in conjunction with cytokines. It is worthwhile to further validate the predictive values of these biomarkers as a tool to select or exclude patients who need more intensive management to rationalize the use of limited resources.

There are several limitations of this study. Biospecimen processing, deconvolution of ^1^H-NMR spectra, software capabilities, quantitative analysis and reporting of contents can differ between service providers. The Nightingale platform, as used in this targeted metabolomic study, predominantly included lipids and lipoprotein-related markers and has been used by a number of large-scale longitudinal cohort studies [11]. Other ^1^H-NMR platforms, including the Bruker Avance IVDr and lifespin NMR, provide proportionately greater numbers of amino acids and other metabolic pathway intermediates in their reporting. It is possible that those other platforms may reveal additional non-lipid metabolomic shifts and targets not seen in this analysis. Second, our results were generated using blood plasma which may differ from those of serum [33]. Depending on the metabolic marker of interest, reproducibility has been shown to be similar between plasma and serum, and a study examining different blood collection tubes found no significant effects of the preservative on lipoproteins [34]. Finally, although this study provides valuable insights into the prognostic utility of specific biomarkers in COVID-19, it is limited by the availability of additional clinical data. Comprehensive clinical characterizations such as detailed patient comorbidities, body mass index (BMI), specific treatments, post-vaccination antibody levels and the use of certain medications like hypolipidemic drugs and corticosteroids were not uniformly available across the patient cohort, limiting our ability to assess the influence of prior immunity on disease severity and outcomes. Future studies should aim to incorporate these elements through a prospective design, ensuring a more comprehensive dataset that allows for a nuanced analysis of the interplay between clinical characteristics, treatment modalities and biomarker efficacy in predicting COVID-19 outcomes.

In conclusion, we demonstrated the tight interaction and prognostic potential of metabolomic and immunologic biomarkers, which may enable patient stratification based on their likelihood of developing severe disease. At present, ^1^H-NMR-metabolomics is scarcely available, the equipment cost is high and specially trained personnel is required to operate the platform. Accessibility of this powerful blood phenotyping technology may improve over time, and allow implementation of the pipeline into the workflow of healthcare facilities to enable rapid, high-dimensional and efficient service. Applications of metabolomics to understand other important infectious diseases are encouraged.

## Figures and Tables

**Table 1:** Characteristics of study subjects.

Characteristics	Total (*n* = 327)	Non-Omicron ^#^ (*n* = 217)	Omicron * (*n* = 110)
Gender			
Female	217	160	57
Male	110	57	53
Age, mean ± SD (range), years	55 ± 17 (19–89)	52 ± 16 (21–85)	61 ± 19 (19–89)
19–60	172	134	38
61–65	39	32	7
66–89	116	51	65
Sampling from onset, mean ± SD (range), days	7 ± 5 (1–33)	7 ± 5 (1–24)	6 ± 5 (1–33)
≤7	212	128	84
>7	115	89	26
COVID-19 severity			
Asymptomatic	14	10	4
Mild	123	66	57
Moderate	91	83	8
Severe	35	28	7
Critical	55	27	28
Fatal	9	3	6
Peak viral load, mean ± SD (range), Ct	23.54 ± 6.57 (11.10–38.97)	24.75 ± 6.76 (11.10–38.65)	21.46 ± 5.67 (11.32–38.97)
≤20	92	50	42
>20	171	116	55
NA	64	51	13
Peak C-reactive protein, mean ± SD (range), mg/L	47.85 ± 76.55 (0.06–487.03)	39.52 ± 64.96 (0.06–487.03)	62.25 ± 91.79 (0.13–466.74)
≤30	188	122	66
>30	112	68	44
NA	27	27	0
25-OH vitamin D, mean ± SD (range), ng/mL	17.69 ± 9.37 (1.44–79.47)	17.01 ± 10.11 (2.78–79.47)	18.74 ± 8.02 (1.44–43.18)
≤12	85	61	24
13–20	97	62	35
21–30	71	31	40
>30	27	17	10
NA	47	46	1
Day from illness onset	7 ± 5 (1–33)	7 ± 5 (1–24)	6 ± 5 (1–33)
≤7 days	212	128	84
>7 days	115	89	26
SARS-CoV-2 mRNA or inactivated vaccine			
No	254	217	37
1 dose	22	0	22
2 doses	45	0	45
3 doses	6	0	6

^#^ Non-Omicron, patients recruited during the first four waves between 2020–2021 before the emergence of Omicron variants. * Omicron, patients recruited during the fifth wave after January 2022, and were all infected with Omicron variants, mainly BA.2. SD, standard deviation; Ct, threshold cycle; NA, data not available.

## Data Availability

After publication, the data will be made available on reasonable request to the corresponding author. A proposal with a detailed description of study objectives and a statistical analysis plan will be needed for assessment of requests. Additional materials might also be required during the process of assessment. Deidentified participant data will be provided after approval by the investigators.

## Supplementary Materials

The following supporting information can be downloaded at https://www.mdpi.com/article/10.3390/metabo14070380/s1: Table S1: Characteristics of study subjects; Table S2: Association of plasma metabolite levels with COVID-19 disease severity; Table S3: Association of plasma cytokine/chemokine levels with COVID-19 disease severity; Table S4: Correlation between plasma cytokine/chemokine and metabolite levels. Only the Omicron group with both cytokine/chemokine and metabolomic data available are included.
